# A semi-automated, KNIME-based workflow for biofilm assays

**DOI:** 10.1186/s12866-016-0676-9

**Published:** 2016-04-06

**Authors:** Katrin Leinweber, Silke Müller, Peter G. Kroth

**Affiliations:** Zukunftskolleg, Universitätsstraße 10, Postbox 216, Konstanz, 78457 Germany; Konstanz Research School Chemical Biology (KoRS-CB), Universitätsstraße 10, Postbox 630, Konstanz, 78457 Germany; Screening Center Konstanz, Universitätsstraße 10, Screening Facility, Konstanz, 78457 Germany; Department of Biology, University of Konstanz, Universitätsstraße 10, Postbox 611, Konstanz, 78457 Germany

**Keywords:** Diatoms, *Achnanthidium minutissimum*, Biofilms, Medium throughput, Diatom-bacteria interactions, Bioassay, KNIME

## Abstract

**Background:**

A current focus of biofilm research is the chemical interaction between microorganisms within the biofilms. Prerequisites for this research are bioassay systems which integrate reliable tools for the planning of experiments with robot-assisted measurements and with rapid data processing. Here, data structures that are both human- and machine readable may be particularly useful.

**Results:**

In this report, we present several simplification and robotisation options for an assay of bacteria-induced biofilm formation by the freshwater diatom *Achnanthidium minutissimum*. We also tested several proof-of-concept robotisation methods for pipetting, as well as for measuring the biofilm absorbance directly in the multi-well plates. Furthermore, we exemplify the implementation of an improved data processing workflow for this assay using the Konstanz Information Miner (KNIME), a free and open source data analysis environment. The workflow integrates experiment planning files and absorbance read-out data, towards their automated processing for analysis.

**Conclusions:**

Our workflow lead to a substantial reduction of the measurement and data processing workload, while still reproducing previously obtained results in the *A. minutissimum* biofilm assay. The methods, scripts and files we designed are described here, offering adaptable options for other medium-throughput biofilm screenings.

**Electronic supplementary material:**

The online version of this article (doi:10.1186/s12866-016-0676-9) contains supplementary material, which is available to authorized users.

## Background

Diatoms (Bacillariophyceae) are a group of highly productive unicellular photoautotrophs and responsible for roughly one fifth of Earth’s primary production [1, 2]. Many diatoms produce and secrete extracellular polymeric substances (EPS) which are a major component of their extracellular polymeric matrices, and which convey motility and substrate adherence [3, 4]. Such aggregates of microbes that are embedded in a matrix of secreted EPS form so called biofilms [5]. Biofouling, the colonisation of man-made structures like e.g. ship hulls or underwater constructions by diatoms and other aquatic organisms may causes considerable maintenance costs [6, 7].

*Achnanthidium minutissimum* is a benthic pennate diatom that appears cosmopolitically in freshwater habitats which may form biofilms on substrata [8]. Carbohydrate secretion and biofilm formation by *A. minutissimum* can be induced by certain bacteria, but also in the presence of substances secreted by the bacteria, indicating that the bacteria induce or even may control biofilm formation by the diatom [9, 10]. In order to quantify biofilm formation as well as to purify and identify bacterial signal substances, a bioassay based on these effects has been established in our laboratory previously [11]. This biofilm assay relies on the staining of diatom cells with crystal violet (CV). This compound is also used for staining of Gram-positive bacteria. It is binding to organic polymers such as polysaccharides and peptidoglycans ([12], *Table 2*). Subsequently, the stain can be extracted with ethanol (EtOH), and quantified photometrically. However, this protocol is not automated, and thus proved to be difficult to apply to medium-throughput screenings, e.g. for biofilm-inducing, bacterial infochemicals or biofilm-relevant mutants.

Inspired by other biofilm assay approaches which include automated steps [13, 14], we aimed to improve the *A. minutissimum* bioassay described above with semi-automated pipetting and absorbance read-out methods. Additionally, we wanted to improve the preparation of sample metadata and biofilm measurement data for analysis, adopting an already widely used tool in nucleotide sequencing and biochemical screening: the Konstanz Information Miner (https://www.knime.org/) (KNIME; [15, 16]). The program is an interactive, visual, modular environment and open platform for the design and execution of data analysis workflows [17]. To the best of our knowledge, no diatom- or biofilm-focussed approach so far is based on KNIME-supported data analyses or shares such workflows. Therefore, the objective of this study is to explain the above-mentioned automation options, and to present compelling evidence for their improvement potential using several examples from a laborious diatom biofilm assay.

## Methods

### Cultivation conditions

Xenic cultures of *Achnanthidium minutissimum* (Kützing) [18] were isolated from epilithic biofilms from the littoral zone of Lake Constance, and axenified as described previously [19]. Both xenic and axenic *A. minutissimum* stocks were cultivated in modified BM (Bacillariophycean medium) in ventilated tissue culture flasks (Sarstedt, Newton, NC, USA). These were stored in a climatised culture room at 16 °C, 50 % humidity and in a day-night cycle of 12 h at 20–50 $\frac {\mu \text {mol~photons}}{\mathrm {m}^{2} \cdot \mathrm {s}}$ and 12 h of darkness. Cultures were subcultivated monthly by scraping cells off the flask bases and transferring 1 mL of the resulting suspension into fresh flasks with ca. 40 mL BM.

Bacteroidetes strain 32 (S32) was cultivated on agar plates prepared from 50 % (v/v) LB [20]. Between monthly transfers to fresh plates, S32 was grown for 5 days at 22 °C in darkness, and was then stored at 4–8 °C. Liquid S32 cultures were prepared by inoculating 50 % LB and shaking at 22 °C and 120 rpm for 3–5 days.

### Human- and machine-readable plate layout worksheets

**Fig. 1:**
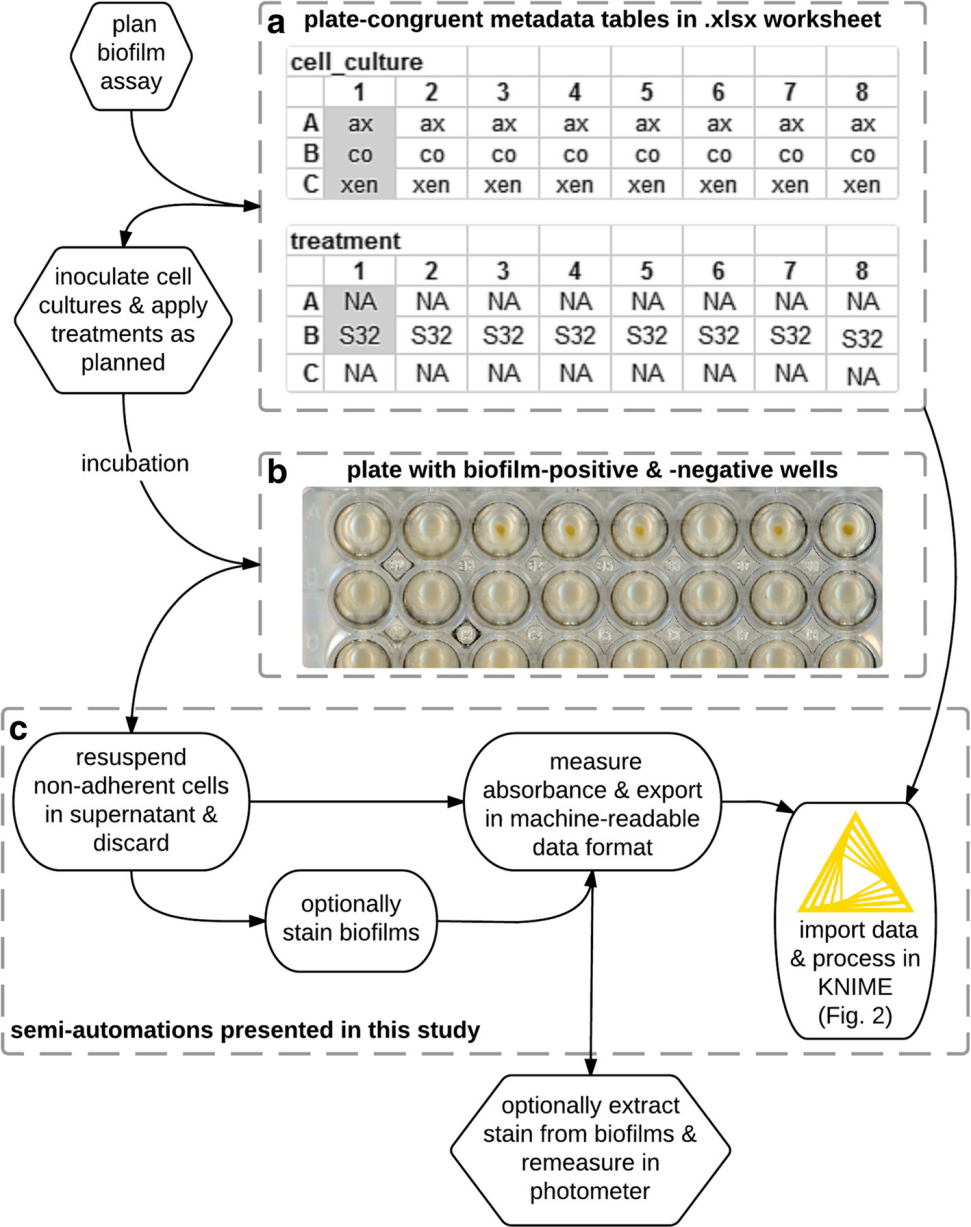
Overview of semi-automated biofilm assay from metadata tables (**a**) to measurement workflow (**c**). *Hexagonal boxes* indicate manual steps of biofilm assay procedure. *Rounded boxes* represent robotised steps. *Dashed, grey-lined boxes* represent main sections of the biofilm assay protocol. **a** Metadata tables in .xlsx worksheet are used to plan the layout of samples on the multi-well plate (see Additional file 1). Table headings represent factors (*cell_culture* & *treatment*), whose levels (respectively, *ax*, *co* & *xen*, as well as *NA* for controls & *S32* for co-cultures) fill the plate-congruent coordinate systems of the tables. *Grey cells* indicates bases for formulas, which help to rapidly fill repetitive tables with for example technical replicates or dilution calculations. **b** Photo of incubated plate, ready for removal of non-adherent cells and absorbance measurement. **c** Robotised measurement preparation (see Table 1), absorbance read-out and data processing from biofilm assay plates (see Additional files 3, 4, 5 and 6)

The first optimisable step of the biofilm assay experiments was the planning of sample layouts in the multi-well plates before their inoculation. Our proposed plate layout scheme is based on Microsoft’s Office Open XML format (.xlsx files), of which we used the worksheets to represent the multi-well plates (Fig. 1a and Additional file 1). These worksheets are both human- and machine-readable. In each sheet, the sample metadata was contained in several plate-congruent coordinate systems which are titles with the metadata type (e.g. culture, treatment, identifiers, etc.). Because our workflow produces data files which are compatible with the R environment for statistical computing, in the terminology of R the table titles are *factors* and the contents are *levels*.

**Table 1:** Overview of electronic pipetting scripts executed by the Viaflo ASSIST robot (http://www. integra-biosciences.com/sites/viaflo\_assist.html)

Step	Mode	Script task	Height/Z	Speed	Follow-up
1.	Custom	Mix & remove cells	54.5 mm	7	Yes
2.	Repeat disp.	Add 200 *μ*L CV	10 mm	3	No
3.	Custom	Remove CV	54.5 mm	3	Yes
4.	Repeat disp.	Add 1 mL H_2_O	10 mm	5	No
5.	Custom	Remove H_2_O	54.5 mm	8	Yes

The KNIME workflow is agnostic towards the number of wells per plate and the number of factors and levels. Therefore, all plate-congruent tables can be adjusted congruently to other multi-well formats by adding or removing rows and columns. More such tables can be added to the worksheets in order to add more metadata to the samples, which can be used later in the data analyses and visualisations. Adding more plates to an experiment was mirrored by adding more worksheets to the same .xlsx file. Spreading the worksheets across different .xlsx files is possible, but may cause more work later when preparing the KNIME workflow.

### KNIME workflow for data processing

**Fig. 2:**
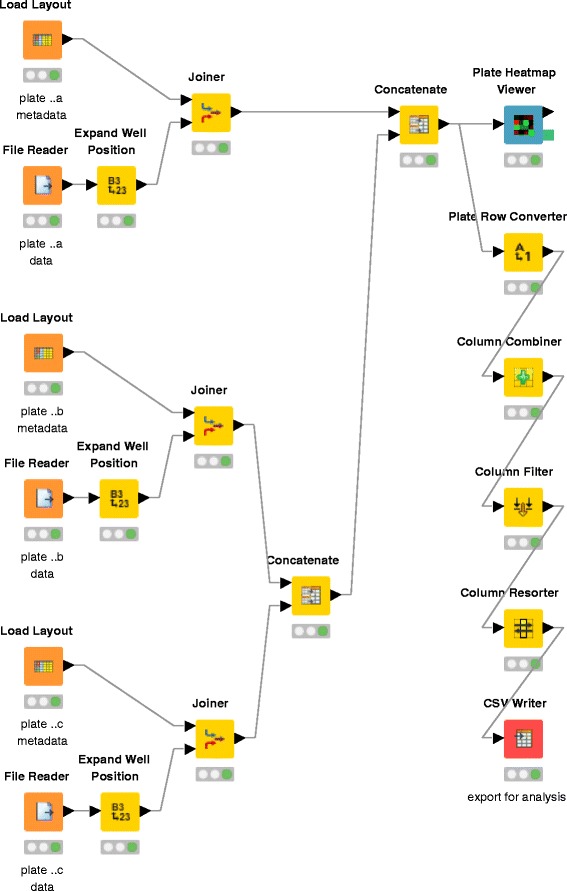
KNIME workflow example for the integration of sample metadata and measurement results for analysis & visualisation. The *green indicator* below each node indicates that the entire workflow has been successfully executed. See Additional file 5 for an importable copy of this workflow, which serves as an interactive tutorial for the methodology presented in this article

Due to the incubation time of the diatom biofilms, about two weeks usually passed between preparing the plate layout worksheets and obtaining the measurement results as described in sections Bioassay experiments and Absorbance read-outs of stained and unstained biofilm assays. In order to link planning documents of an experiment with the final data analyses and results visualisations, we designed a data processing workflow that unifies multiple files for further analysis (Fig. 2 and Additional file 5). The workflow was implemented in the Konstanz Information Miner (KNIME) (https://www.knime.org/knime) v3.1.1 [17] using freely available functions and nodes, particularly the HCS-Tools (https://github.com/knime-mpicbg/HCS-Tools) extension [21].

Before execution of this workflow, three manual adjustments have to be made. Firstly, file path and name of the layout worksheets in .xlsx format (see Fig. 1a and Additional file 1) were selected for each plate through the respective Load Layout nodes. Secondly, once the corresponding absorbance data files were obtained, they were selected through the respective File Reader nodes. Lastly, the machine-readable export target file has to be defined in CSV Writer.

More plates can be accommodated by inserting more groups of Load Layout, File Reader, Expand Well Position, Joiner and Concatenate nodes into an existing connection between a Joiner and a Concatenate node by copying, pasting and reconnecting the relevant nodes. Thus, the researcher can quickly expand the provided minimal example (see Fig. 2) to the desired number of plates, accommodating any number of biological or technical replicates at a time and/or experiment repetitions over time, as long as the tree-like scheme is preserved.

**Fig. 3:**
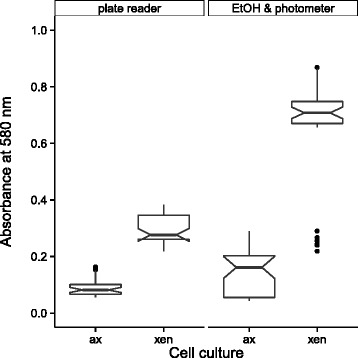
Comparison of photometer- and plate-reader-based absorbance measurements of CV-stained *A. minutissimum* biofilms. Biofilms stained with CV were first measured in the wells with a plate reader (*left facet*). Afterwards CV was extracted with EtOH, transferred into photometric cuvettes and re-measured in a spectrophotometer (*right facet*). Axenic (ax) and xenic (xen) wells represent the negative and positive controls in this bioassay (non-adherent and adherent cells, respectively; *N* = 36). *Boxes* represent the first and third quartiles. Whiskers extend to the lowest and highest value that lies within 1.5-fold of the inter-quartile range (IQR). *Black dots* are extreme values that lie outside the IQR. *Black center lines* represent medians. *Notches* indicated the range of $1.58 \cdot IQR / \sqrt {N}$ (ca. 95 % confidence interval) around the medians. For a more detailed explanation of box-and-whisker plots and their variants, we recommend reading [40]. See also Additional file 7 for R code and data used to plot this and the following figures

**Fig. 4:**
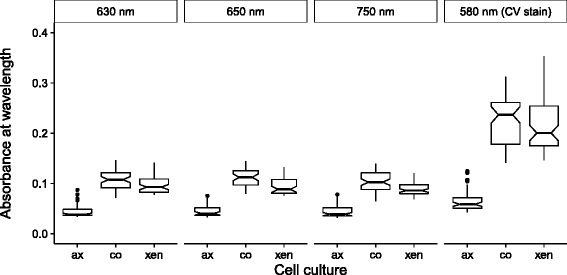
Detection of *A. minutissimum* biofilms with a plate reader before and after staining with CV. Absorbance was measured at wavelengths 630, 650 and 750 nm before staining the biofilms with crystal violet (CV), and at 580 nm after the staining. Negative, axenic controls; biofilm-positive, xenic controls; co-culture of axenic *A. minutissimum* and Bacteroidetes strain 32; *N* = 48 to 64 wells per culture type, spread across 4 plates, across 2 independent experiments

**Fig. 5:**
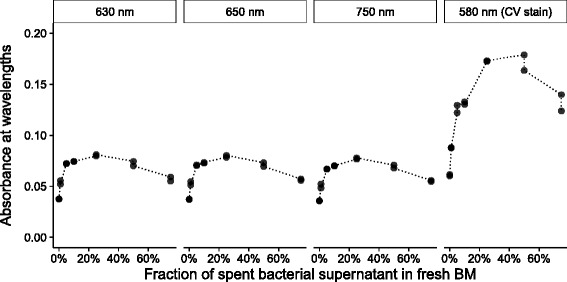
Detection of differently strong *A. minutissimum* biofilms before and after staining with CV. Bacteroidetes strain 32 supernatant was centrifuged and sterile-filtered from three 7 d old glcBM liquid cultures with OD_600_ of 0.25. Biofilm induction was highest around 25–50 % spent bacterial medium (v/v) as shown in Figure 3A of [11]

Based on the exported .csv, downstream analysis and visualisation of the KNIME-processed data (Figs. 3, 4 and 5) was conducted in a separate work step in RStudio v0.99 Desktop Open Source Edition (http://www.rstudio.com/products/rstudio/) with the ggplot2 (http://ggplot2.org/) package v2.0.0 (https://github.com/hadley/ggplot2/releases/tag/v2.0.0) [22] and R v3.2.3 [23] (see Additional file 6).

### Bioassay experiments

Bioassays were prepared in 48-well plates (#677180, Cellstar (https://shop.gbo.com/de/germany/articles/catalogue/article/0110_0040_0110_0010_0010/13533/); Greiner Bio-One, Frickenhausen, Germany) by scraping both axenic and xenic *A. minutissimum* cultures off the flask bottoms and washing the cells once with fresh BM to remove residues of the spent cultivation medium (see Additional file 2). Axenic cells in this stock suspension were counted in a Thoma chamber in order to calculate the cell concentrations. After extracting chlorophyll in 5 % MeOH and 95 % acetone according to Jeffrey & Humphrey [24] from both diatom cultures, their washed suspensions were independently adjusted to $10^{6} \frac {\text {cells}}{\text {mL}}$. For that purpose, both cultures were assumed to contain equal amounts of chlorophyll per cell. This stock suspension of 50 *μ*L (containing 5·10^4^ washed cells) was transferred into each well, as pre-recorded in the worksheets discussed in section *Human- and machine-readable plate layout worksheets*.

Treatments were administered within minutes of pipetting the diatom cultures into the multi-well plates. For co-culture experiments of the axenic diatom with S32, suspensions of the bacterial cells were prepared by centrifuging the LB liquid cultures at 5−10·10^3^ g for 5–10 min and washing them with BM supplemented with 10 mM glucose (glcBM). The washed bacterial cells were resuspended in BM to an OD of 0.1 and 5 *μ*L of that suspension was added to the wells with the axenic *A. minutissimum* cultures. For supernatant experiments, different volumes of sterile-filtered bacterial supernatant were added to the wells. This supernatant was harvested from S32 cultures in glcBM and filtered through 0.2 *μ*m pores (Sarstedt AG, Nürmbrecht, Germany). In either case, each well was filled with fresh BM to a final volume of 500 *μ*L. Negative control wells contained untreated axenic *A. minutissimum*, while xenic cultures served as biofilm-positive controls. Bioassay plates were sealed with Parafilm (Bemis, Neenah, WI, USA) and cultivated for 10–12 d as described for the stock cultures in section *Cultivation conditions*.

### Robotised biofilm quantification

We evaluated the utility of robotised pipetting for the removal of non-adherent cells and medium, as well as for the staining of the remaining biofilms with crystal violet (CV) as visualised in Fig. 1c. VIALINK Pipette Management Software (http://www.integra-biosciences.com/sites/vialink.html) was used to program *custom* scripts. *Repeat Dispense* scripts were programmed on a 1250 *μ*L Viaflo Electronic Pipette (http://www.integra-biosciences.com/sites/viaflo_pipettes.html). The latter was used with six 1250 *μ*L pipette tips for all pipetting steps, along the long axis of the 48-well plate. We tested using eight tips and pipetting along the short axis of the plate, but found that they did not reliably align with the wells. Both script types (see Table 1) were executed with the pipette connected to the Viaflo ASSIST (http://www.integra-biosciences.com/sites/viaflo_assist.html) robot (INTEGRA Biosciences AG, Switzerland).

The crucial, optimisable step for removing non-adherent cells is the mixing step. For our *A. minutissimum* cultures, mixing was optimised in terms of immersion depth (54.5 mm; see Table 1), mixing volume (400 *μ*L), speed (7) and cycles (7) at room temperature, so that axenic cultures were resuspended while the xenic biofilms were not disturbed. These settings have to be adjusted for other organisms, in order to accommodate different biofilm thicknesses, strengths of adherence, etc.

The plate reader device Tecan Infinite F500 (http://lifesciences.tecan.com/products/reader_and_washer/microplate_readers/infinite_f500) (Tecan Group Ltd., Switzerland) was used at room temperature to measure biofilm absorbances. For that purpose, a custom method (see Additional file 4) was implemented in the Magellan (http://lifesciences.tecan.com/products/software/magellan_data_analysis_software) dataa analysis software v7.1. It took absorbance measurements at 16 evenly spread points in a filled circle with a 1.7 mm distance to the well edge. The absorbances of unstained biofilms were read out at 630, 650 and 750 nm. These wavelengths were chosen according to Jeffrey & Humphrey [24] for the chlorophyll c absorbance maximum (630 nm), for a compromise between the chl a maximum and an available filter (650 nm), as well as for the light scattering due to cells (750 nm). After measuring the absorbances of the unstained biofilms, they were incubated for 1 min with 200 *μ*L of a 1:100 dilution of the crystal violet (CV) staining reagent described previously [25]. The CV solution was removed by pipetting as described above. Afterwards, sterile-filtered tap water was added to the wells for washing, and removed after 1 min. The plate-reader was used again to measure the absorbance at 580 nm. Finally, the Magellan method ensured the data export into machine-readable .asc files for subsequent input into the KNIME workflow.

For comparison of the above described robotised biofilm quantification with the manual protocol established previously [11], CV was extracted from the wells after plate-reading. This was done by pipetting 1 mL EtOH up and down several times and the resulting CV solution was transferred into a quartz cuvette. The absorbance was measured at 580 nm in an Ultrospec 2100 pro (http://www.thomassci.com/Instruments/Spectrophotometers/_/0A56FFDB-A8B9-473E-99CF-960599F301B7) UV/visible spectrophotometer (Biochrom Ltd. Cambridge, UK (http://www.biochrom.co.uk/contact/)).

## Results and discussion

### Human- and machine-readable data processing

Regarding our use of the plate layout worksheets and KNIME, we found that using such software for managing experimental workflows is advantageous [26, 27]. This results from the data processing being reviewable independently [28], reproduced by other researchers, as well as prepared and tested with example data or initial measurement data. We also found KNIME useful for tracking the progress of experiments because all nodes indicate their status (traffic light symbols in Fig. 2). This becomes particularly useful for complex experiments during which replicate data is obtained at different times and/or after long incubation times (e.g. seasonal repetitions [29]).

Preparing plate layouts in .xlsx-compatible programs enabled the calculation and planning of dilution series, and even randomisations. However, the potential benefit of randomised sample placement should be evaluated on a case-by-case basis against the increased workload and the risk of pipetting errors during manual inoculation. This risk can be mitigated by keeping dilution series in their inherent order, but pipetting them in alternating directions across the plate (pseudo-randomisation). The worksheets should obviously be prepared before the inoculation, in order to be useful as pipetting guides at the bench. However, they can be updated at any time to reflect pipetting errors, changes of the experimental plan, or the sample sets, etc.

The KNIME workflow may be improved by integrating such scripts, so that data analysis and visualisation can be started immediately with the first absorbance data file. Because inputting the paths of the corresponding layout worksheets and absorbance data files into the nodes is repetitive, it may be desirable to automate the detection and pairing of corresponding files in large numbers. Such an automation may be possible using KNIME’s Read XLS Sheet Names, StringManipulation, flow-variables and loops (personal communication, Patrick Winter).

### Robotised biofilm quantification

Regarding the robotised liquid removal steps, we found that they could not be conducted reliably at this immersion depth, because notable residue volumes remained in the wells. Immersing the pipette tips further risked scratching the biofilms, so we instead used the electronic pipettes manually, tilting the tips into the well edges. Despite this manual removal of liquids, the time requirement and work intensity per plate could be reduced considerably from ca. 30–45 min of intense manual pipetting [11] to ca. 10–15 min of intermittent electronic pipetting and device operations.

A read-out of the EtOH-filled wells after CV extraction was possible, but similarly to a report by Rasmussen & Østgaard [30] (*Discussion* | *Experimental Systems*), the meniscus of the solution distorted the measurements in a ring-shaped area around to the well edges. As smaller well sizes may exacerbate this problem, we refrained from using 96-well plates. Manual removal of residual liquid with finer pipette tips, or a separate drying step might alleviate this risk, but would extend the time for measurements.

Our workflow can be adopted easily to other organisms and biofilm assays by adjusting the plate-reading method (Additional file 3; particularly the number of the measurements, as well as the chosen wavelengths) in Tecan’s Magellan software. One potential improvement is the randomisation of the measurement positions in the wells [31], but this was not yet available in our Magellan version.

The bottleneck of this workflow is the number of samples and plates that can reasonably be inoculated manually. Dispensing Bacillariophyceaen medium (BM) and *A. minutissimum* with a stepper or multi-channel pipette requires only a few minutes per 48-well plate, but the application of different treatments (e.g. S32 co-cultivation or its supernatant extracts, or compounds at different dilutions, etc.) easily multiplied that time per plate. To remove this bottleneck, further robotisation protocols similar to those reported previously [32] are required. For that purpose it will be necessary to either add control cultures for that protocol’s semi-sterile nature, or to optimise it towards fully sterile conditions. As a next step, a robotised *A. minutissimum* and S32 co-cultivation system could be connected to high-resolution mass spectrometry in order to elucidate the nature of biofilm-inducing substance [11, 32].

### Absorbance read-outs of stained and unstained biofilm assays

The initial proof-of-concept of the above-described measurement protocol was conducted with non-adherent, axenic (ax) and biofilm-forming, xenic (xen) *A. minutissimum* cultures (Fig. 3). Using the plate reader resulted in lower absolute absorbances for both culture types. The spread of data read-outs from axenic replicates was about half of those from the EtOH- and photometer-based measurements. For xenic biofilms, the spread was only 25 % smaller, but the plate reader measured lower absorbances. Nevertheless, the absorbance read-outs still were clearly distinct between axenic and xenic samples.

The EtOH- and photometer-based method [11] resolved the differences of biofilm intensities better. However, xenic replicates with strong deviation below the median absorbance could result from drying of the biofilms before staining or before the extraction of the stain with EtOH. Axenic replicates with strong deviation above the median absorbance could readily result from small crystal violet residues getting carried into the EtOH extraction. Therefore, the EtOH- and photometer-based method risks false measurements in both controls. This previous method is only feasible for the quantification of small sample sets. The faster plate-reading was less error-prone and more reproducible. It still allows the EtOH- and photometer-based method to be appended in case additional verification of unclear read-outs is required.

The next step of our proof-of-concept deals with the detection of unstained biofilms at 630, 650 and 750 nm (Fig. 4). Median absorbances were significantly distinct between the axenic, non-adherent control and the biofilm-forming co- and xenic cultures in any case. The total spreads of data from the axenic controls overlapped only slightly with those of the two biofilm-positives sample types (co- and xenic culture). The median absorbances after staining with CV were more distinct, and the data spreads did not overlap, although those of the biofilm samples were larger. In summary, the medians and spreads of data show that the distinctiveness of unstained biofilm-free and -containing wells was reflected by the absorbances on three different wavelengths as well as by the CV-specific wavelength.

The similarity of biofilm absorbances read-outs at 630, 650 and 750 nm confirms other biofilm measurements conducted at wavelengths of 680 nm [33] and 600 nm [14]. The latter study also involved staining with CV of bacterial biofilms in multi-well plates, in order to test combinatorially produced surface coatings for anti-fouling capabilities. The similarity of technical set-ups suggests that our KNIME-based workflow can be applied beyond diatom biofilm research. In another photometric approach [13] monitored biofilm formation of the diatom *Planothidium* sp. in a sterile incubator. They detected the growth of unstained biofilms by attenuation of the visible light spectrum around a peak of 600 nm in real-time. The common result of those cited with our study is that diatom and bacterial biofilms can be detected between 580 and 680 nm. This suggest that any wavelengths within this range is suitable. Moreover, the cited biofilm assays were conducted with less than ten replicates. Our workflow helps to handle more biological and/or technical replicates at a time, experiment repetitions over time, and/or repetitions by different researchers. All these are key to the statistical power of studies and help to reduce the risk of false research findings [34], but increase the workload considerably. Our workflow decouples the number of replicates and repetitions from the data processing and analysis workload.

The lower absolute absorbances of unstained biofilms at 630, 650 and 750 nm (Fig. 4) can be explained by the detection of only adherent cells. CV stains EPS structures like stalks and capsules in addition to the cellular polymeric structures [12], and thus contributes to the high absolute absorbances at 580 nm. EPS are important for the surface attachment of microbes and are a logical requirement for biofilm formation [3, 4]. Our protocol supports the staining where necessary, but the elimination of CV staining from workflows does not prohibit the biofilm detection. CV-free workflows can be desirable, because the stain may increase the risk of false-positives, and is classified as toxic to aquatic life [35].

As a final assessment of our proof-of-concept, we tested the biofilm induction in axenic *A. minutissimum* by addition of sterile-filtered S32 supernatant. We used the same measurement set-up as described for Fig. 4. All absorbance curves peak between 10–50 % (v/v) supernatant (Fig. 5). Absorbances of unstained biofilms measured by plate-reader at 630, 650 and 750 nm had lower absolute differences. At those wavelengths, the unstained biofilms had the absorbance maxima at 25 % supernatant. After staining with CV and measurement at 580 nm, the maximum shifted to the range of 25–50 %.

The shapes of all biofilm-induction curves (Fig. 5 agree with analogous curves determined previously with the EtOH-extraction protocol ([11], Fig. 3A therein). This demonstrates that the less time-consuming protocol proposed in the present study is able to detect differently strong, unstained biofilms, as already discussed for the light-attenuation-based evidence reported previously [13]. In addition to the faster, non-invasive biofilm detection, our workflow leaves two opportunities to increase the absorbance resolution for unclear samples: (1) Read-out of stained biofilms can easily be integrated; (2) EtOH extraction of the stain and photometric measurement can be appended as well.

To summarise, the three proof-of-concept steps confirmed the results of previous studies and highlighted several simplification and automation options. These options are applicable for biofilm assays in general, and specifically enhance the utility of *A. minutissimum* in environmental monitoring. This is relevant, because this diatom has been used previously as a bioindicator of heavy metal pollution [36, 37]. That application relied on the detection of frustule deformations – an approach that has been questioned by Lavoie et al. [38] who observed the valves often settling in girdle view, which hindered the quantification of frustule deformations in this diatom species. Using *A. minutissimum* as an example, we present the simpler, robotised proxy measurement of the presence or absence of cells as a means to detect biofilms. Thus, we demonstrate here an improved in vitro variant of *in situ* “stable platforms”, which Amin et al. [39] recommended for the study of diatom-bacteria interactions.

## Conclusions

Because we show in several examples that staining with crystal violet is not strictly required for the detection of *Achnanthidium minutissimum* biofilms, our workflow may enable a broad range of existing plate-reading set-ups to be used for biofilm assays, while reducing chemical waste. By demonstrating the successful implementation of human- and machine-readable data structures and processing workflows in the free and open source software KNIME, we offer generally useful options for the standardisation, up-scaling and replication of biofilm assays that utilise diatoms, bacteria or other microbes. Particularly the *A. minutissimum* bioassay, but also other laborious assays, may thus become applicable for use in medium-throughput screening of chemicals or mutant strains for biofilm-inducing or -inhibiting effects, due to the considerable reduction of measurement and data processing workload. This will stimulate the use of biofilm assays in environmental and anti-biofouling research. Moreover, since the KNIME workflow is agnostic to the type of measurement and sample preparation, its basic concept of integrating planning documents and measurement results is applicable to any other experiment with multi-well plates.

## Availability of data and materials

The dataset supporting the conclusions of this article is included as Additional file 7. Other additional files pertain to the methodology described in this article. Please note that TAR files need to be unpacked (e.g. with 7-Zip (http://7-zip.org) before import into the respective programs.

## Additional files

Additional file 1 Plate layout template for recording sample placement in multi-well plates and merging metadata with measurements in KNIME. See Fig. 1 for illustration. (XLSX 24.4 kb)

Additional file 2 Detailed instructions for the manual preparation of the *Achnanthidium minutissimum* bioassays, based on the protocol by Windler et al. [11]. (PNG 991 kb)

Additional file 3 Viaflo (http://www.integra-biosciences.com/sites/viaflo_pipettes.html) electronic pipetting scripts to successively remove cells, crystal violet staining solution and wash water after steps 1, 3 and 5 (Table 1). See Fig. 1 for experimental context and Vialink’s (http://www.integra-biosciences.com/sites/vialink.html) built-in help for importing instructions. (TAR 35.5 kb)

Additional file 4 Plate-reading method for Tecan’s Magellan software. See section *Robotised biofilm quantification* for details. (MTH 15.6 kb)

Additional file 5 Importable workflow for the KNIME Analytics Platform to demonstrate the merging of sample metadata (plate-layout-template.xlsx) and Magellan-measured absorbance data (.asc files). See Fig. 2 for illustration. Please note that importing will return an error initially, because the file paths can not match, and have to be corrected as described in section *KNIME workflow for data processing*. In case of the File Reader nodes, this correction should be conducted with the option Preserve user settings for new location activated. If forgotten, and if the data preview shows a column filled with question marks, please right-click on that column and activate the option DON’T include column in output table. Traffic light symbols below the nodes will indicate whether corrections are still necessary (red), or whether the nodes can be executed (yellow). Upon execution of this workflow, data files are read in and the Expand Well Position nodes ensure equal formatting of the sample metadata and measurement results according to the well coordinates (defined in the .xlsx file and present in the .asc files). Joiner combines these tables per row, discarding incongruencies between plate layouts and measurement data. Concatenate progressively merges two plates’ data tables into one. Plate Heatmap Viewer provides a visual comparison of the data processing result with the visual impression of a plate. In particular, the expected locations of biofilm-negative and -positive controls are easily discernible. In the concatenated table, Plate Row Converter and Column Combiner regenerate the alphanumeric well coordinates so that the data and visual impression of individual wells can be compared. Column Filter and Column Resorter exclude obsolete coordinate metadata and pre-format the remaining table for export by CSV Writer. (TAR 65.0 kb)

Additional file 6 R code to demonstrate the plotting of KNIME-processed data. Please note that due to a randomisation function in the plate layout .xlsx file, editing the latter and running the KNIME workflow and this script again may produce a plot with different assignments of data points to the levels X, Y and Z. (R 1.22 kb)

Additional file 7 R code and data (.csv format) used to produce the plots in this article. (TAR 45.0 kb)
