# Supplementary figures and images for: A semi-automated, KNIME-based workflow for biofilm assays
Source: BMC Microbiol. 2016 Apr 6;16:61. doi: 10.1186/s12866-016-0676-9 (PMC4823873; doi:10.1186/s12866-016-0676-9)

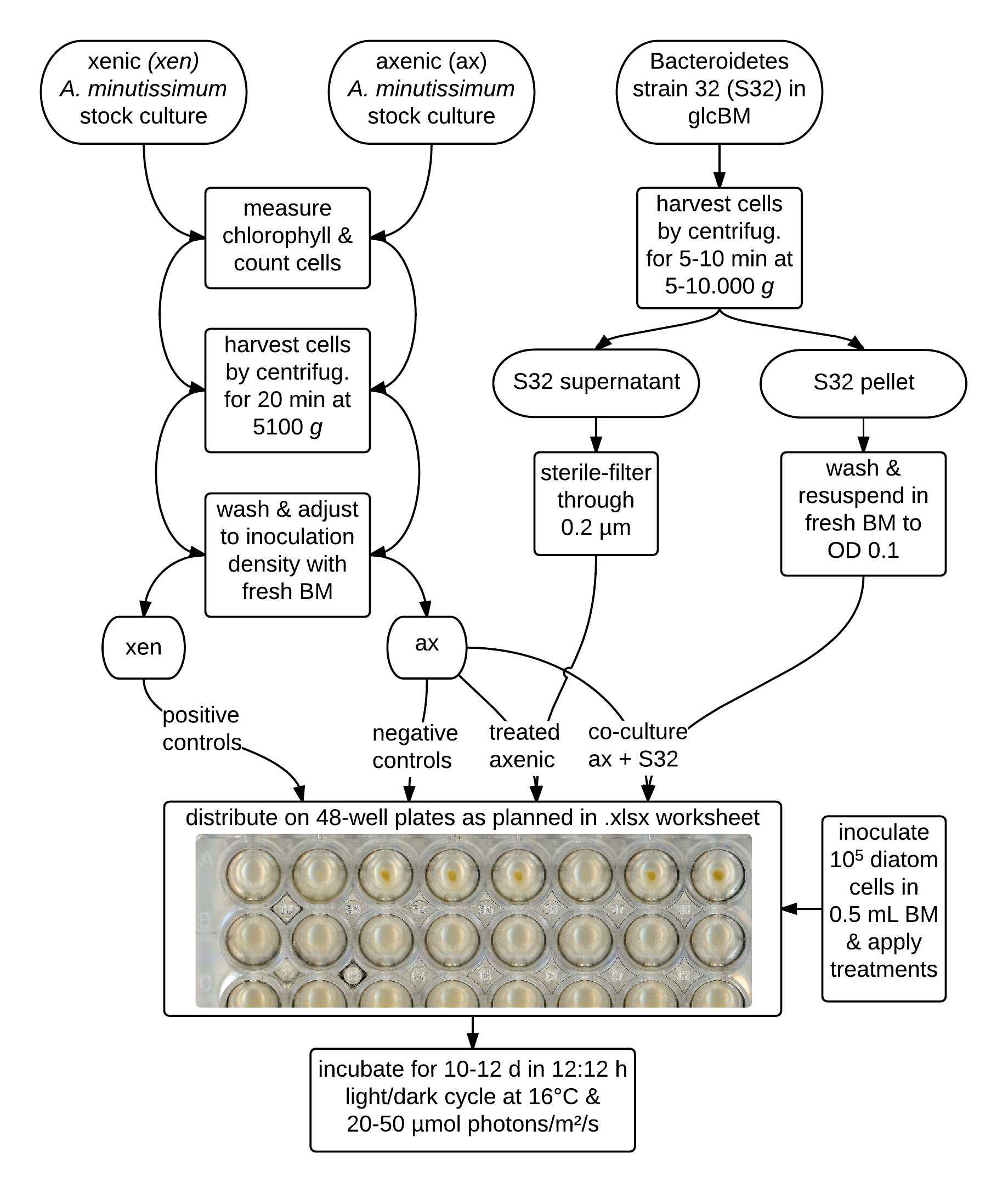

Supplement: Additional file 2 — Detailed instructions for the manual preparation of the Achnanthidium minutissimum bioassays, based on the protocol by Windler et al. [11]. (PNG 991 kb) [file 12866_2016_676_MOESM2_ESM.png]
